# HIV- 1 lentivirus tethering to the genome is associated with transcription factor binding sites found in genes that favour virus survival

**DOI:** 10.1038/s41434-022-00335-4

**Published:** 2022-05-05

**Authors:** Saqlain Suleman, Annette Payne, Johnathan Bowden, Sharmin Al Haque, Marco Zahn, Serena Fawaz, Mohammad S. Khalifa, Susan Jobling, David Hay, Matteo Franco, Raffaele Fronza, Wei Wang, Olga Strobel-Freidekind, Annette Deichmann, Yasuhiro Takeuchi, Simon N. Waddington, Irene Gil-Farina, Manfred Schmidt, Michael Themis

**Affiliations:** 1 Department of Life Sciences, College of Health, Medicine & Life Sciences, Brunel University London, Uxbridge, UK; 2 Testavec Ltd, Queensgate House, Maidenhead, UK; 3 Department of Computer Science, College of Engineering Design and Physical Sciences, Brunel University London, Uxbridge, UK; 4 Genewerk GmbH, Heidelberg, Germany; 5 University Heidelberg, Medical Faculty, Heidelberg, Germany; 6 Institute of Environment, Health and Societies, College of Business, Arts and Social Sciences, Brunel University London, Uxbridge, UK; 7 Centre for Regenerative Medicine, The University of Edinburgh, Edinburgh, UK; 8 Division of Infection and Immunity, University College London, London, UK; 9 Division of Advanced Therapies, National Institute for Biological Standards and Control, Potters Bar, UK; 10 Gene Transfer Technology, EGA Institute for Women’s Health, University College London, London, UK; 11 MRC Antiviral Gene Therapy Research Unit, Faculty of Health Sciences, University of the Witswatersrand, Johannesburg, South Africa; 12 Department of Translational Oncology, NCT and DKFZ, Heidelberg, Germany; 13 Division of Ecology and Evolution, Department of Life Sciences, Imperial College London, London, UK

**Keywords:** Cancer, Stem-cell differentiation, Genetics

## Abstract

Lentiviral vectors (LV) are attractive for permanent and effective gene therapy. However, integration into the host genome can cause insertional mutagenesis highlighting the importance of understanding of LV integration. Insertion site (IS) tethering is believed to involve cellular proteins such as PSIP1/LEDGF/p75, which binds to the virus pre-integration complexes (PICs) helping to target the virus genome. Transcription factors (TF) that bind both the vector LTR and host genome are also suspected influential to this. To determine the role of TF in the tethering process, we mapped predicted transcription factor binding sites (pTFBS) near to IS chosen by HIV-1 LV using a narrow 20 bp window in infected human induced pluripotent stem cells (iPSCs) and their hepatocyte-like cell (HLC) derivatives. We then aligned the pTFBS with these sequences found in the LTRs of native and self-inactivated LTRs. We found significant enrichment of these sequences for pTFBS essential to HIV-1 life cycle and virus survival. These same sites also appear in HIV-1 patient IS and in mice infected with HIV-1 based LV. This in silco data analysis suggests pTFBS present in the virus LTR and IS sites selected by HIV-1 LV are important to virus survival and propagation.

## Introduction

LV have been engineered extensively for efficient and safe therapeutic gene delivery. VSV-G pseudotyped HIV-1 based vectors are particularly well suited for this as they have been shown to infect a broad range of cell types effectively and achieve permanent gene transfer. Following infection and entry into the cell, reverse transcription converts vector RNA genomes into double-stranded cDNA for assembly with cellular proteins [1] into PICs that associate with host chromatin to facilitate integration [2–5].

Clear differences exist in IS selection by retrovirus vectors (RV), that appear to target promoter regions, in contrast to LV that favour the transcription unit of the gene. Integration is semi-random, and genes involved in proliferation, development and differentiation are believed to be favoured [4–7]. Importantly, both RV and LV have been shown to cause insertional mutagenesis and therefore, understanding IS choice is of utmost importance for safe vector design. Tethering of LV to the host genome has been demonstrated to involve the integration complex and several cellular proteins are known to interact with the viral integrase [8–10]. Importantly, HIV LV tethering is believed to be mediated by PSIP1/LEDGF/p75 [4–7, 11, 12] and depletion of PSIP1/LEDGF/p75 significantly reduces HIV integration. However, because HIV-1 IS profile remains semi-random this indicates alternative factors support IS preference [13]. While other sites may influence IS, the LTR is known to be vital in binding to the viral integrase for integration within the host genome [14]. The potential role of transcription factors (TF) in tethering of MLV is supported by the finding that interaction between the MLV integrase and the enhancer in the LTR U3 is important for insertion near specific genomic pTFBS [15]. MLV PIC tethering to pTFBS has been postulated as an important mechanism that promotes viral survival and propagation by enabling TFs to bind gene targets involved in viral transcription [15]. LV, however, is believed not to integrate near to pTFBS after high resolution mapping of IS in haematopoietic cells [16]. Several known TF bind to the HIV-1 genome, for example, TNF-α activation of HIV-1 transcription in chronically infected T-cells requires binding of the NFκB TF specifically to the U3 in the LTR [17]. The HIV life cycle also uses LTR binding sites for c-myb [18] and AP1 [19] TFs to support viral transcription, latency, and infection of non-activated T- cells [20–24]. Several genes important to LV propagation have also been found associated with cancer [25–30]. To understand more clearly the risk for LV to target TFBS that are found in cancer genes, we mapped pTFBS sequences close to IS using a small sequence window of 20 bp around these sites and matched these with the identical pTFBS sequences located in the LV LTR.

Human induced pluripotent stems cells (hiPSC) reprogrammed from somatic cells have the ability to be differentiated to several derivative cell types including HLC which have been used widely for human disease modelling [31–36]. iPSCs have clinical relevance with these cells used in correction of genetic diseases [37–39]. iPSCs and HLCs have been comprehensively characterised as representative of liver-like cells at the genetic and functional level, with these derivatives more closely aligned to in vivo hepatocytes than liver cell lines [40]. Progress has been made in maturation of HLCs to represent a mature phenotype, including growth of spheroid cultures to more closely represent the in vivo microenviroment [40–43]. As RV and LV vectors show preference for integration in highly expressed genes, hiPSC and their derivatives, that express multiple genes involved in developmental and differentiation stages, would be useful to study LV IS selection in genes important to controlling normal cellular behaviour.

In this report, hiPSC and their HLC derivatives were used for infection by HIV-1 LV. We compared LV with native LTR and self-inactivating (SIN) configurations to identify pTFBS targeting, especially since the latter is used for gene therapy. In addition, we aligned these sites in IS of infected hiPSC and HLC by both LV LTR configurations to pTFBS identified in the sequences of the LTRs and found a high degree of similarity. These sites also closely match with pTFBS also present in the IS chosen by HIV-1 in infected patient T-cells and in mice infected with SIN LV. Furthermore, we confirm enrichment of pTFBS that are important for virus survival that also associate with cellular proliferation. These findings imply that further modification of the LTR may avoid these IS targets to achieve safer LV site selection.

## Results

### Identification of pTFBS in LV IS in infected iPSC and HLC

iPSCs were recovered from frozen storage and expanded for differentiation assays. These cells were fully characterised as pluripotent and HLC derivatives before gene transfer to ensure cell identity during these experiments, as described previously [42]. Cells were determined as viable and positive for infection via flow cytometry and PCR analysis and retained their pluripotency and differentiation characteristics.

**Fig. 1:**
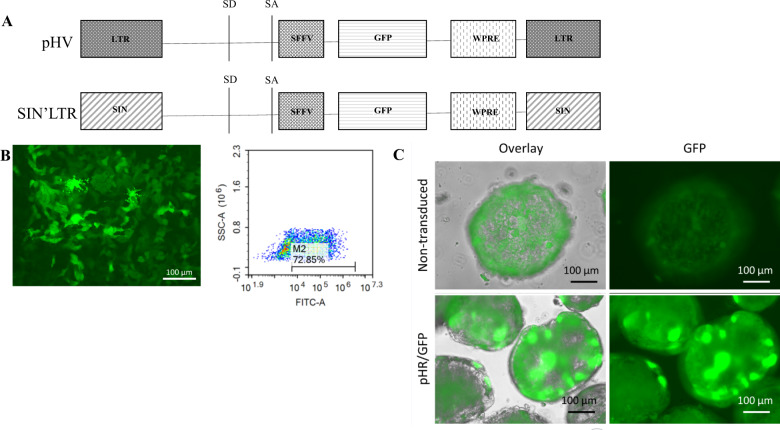
LV infection of in vitro iPSCs and HLCs. **A** Schematic diagram of LV vectors. pHV (native LTR) and SIN’LTR vectors used as reference standards for in vivo and in vitro assays. Both vectors carry an eGFP gene under an internal SFFV promoter. Cells were characterised as pluripotent and liver cells respectively. **B** P106 cells infected with pHR LV. P106 iPSCs were infected with pHR LV at a MOI of 20, with live cell fluorescent microscopy shown 72 h after infection. **C** HLCs were infected with pHR LV vectors (MOI 20). Infected iPSCs were quantified by flow cytometry to express 90% GFP and HLC spheroids were viewed under live cell fluorescent microscopy to show 85% GFP expression in comparison to brightfield images. Example flow plot after iPSC pHR LV infection shown.

**Fig. 2:**
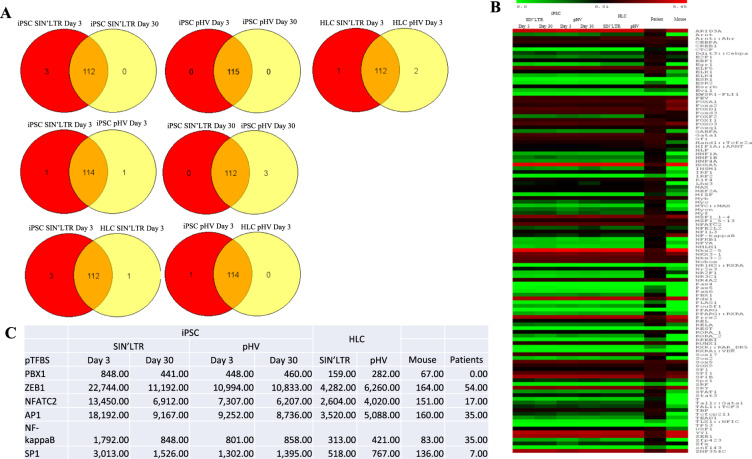
pTFBS identified in in vitro and in vivo LV infected samples. **A** pTFBS mapped from IS in SIN’LTR or native LTR LV infected iPSC had 97% and 100% similarity across the harvested time points and 98% and 97%, respectively between each LV. pTFBS in HLC transduced by SIN’LTR and pHV were 97% in common. Comparison between iPSC and HLC samples indicated 97% (SIN’LTR) and 99% (pHV) of pTFBS identified were in common. **B** Heat map of the relative number of species specific pTFBS identified near IS in in vitro iPSC and HLC infected with SIN’LTR or pHV (native LTR) LV, HIV patients (native LTR) and in vivo mouse (SIN’LTR) samples. pTFBS sequences identified within 20 bp of a particular IS are quantified according to the scale bar shown above the heat map. The proportion of all IS sequences associated with a particular pTFBS in a sample was calculated and expressed as a percentage. The pTFBS are listed in alphabetical order. Patterns can be observed across the in vitro and in vivo data sets. **C** The quantity of pTFBS identified specific for TF known to be involved in HIV lifecycle across in vitro and in vivo samples. Over time in iPSC infected samples, a greater decrease in sequence identity was observed over time in SIN’LTR compared to pHV suggesting the native LTR is driving clonality for IS genes known to be involved in HIV life cycle.

HIV-1 LV carrying the native LTR (pHV) or SIN’LTR configurations driving GFP were used to infect iPSC and their HLC derivatives (Fig. 1). We observed HLC spheroids are heterogenous by light microscopy and therefore postulate this may influence LV IS choice. iPSCs were harvested 3 or 30 days after infection and HLC were harvested 3 days post infection. IS profiling via LAM-PCR [44, 45] identified multiple IS in all infected cells (Table 1). Each IS was identified using the UCSC BLAT genome browser (http://genome.ucsc.edu) alignment to the human genome (hg38 build). The majority of IS were identified within gene bodies. pTFBS were mapped 20 bp either side of each site using oPOSSUM v3.0 Single Site Analysis (http://opossum.cisreg.ca/). Matching pTFBS were found across all samples with regards to timepoint of infection, vector configuration and cell type (Fig. 2 and Table 1).

**Table 1:** Quantity of insertion sites identified and mapped pTFBS across in vitro and in vivo samples.

	iPSC				HLC		Patient	Mouse
	SIN’LTR		pHV					
	Day 3	Day 30	Day 3	Day 30	SIN’LTR	pHV		
IS	118 701	60157	121 160	55 753	22 874	34 142	525 342	193
pTFBS	115	112	115	115	113	114	78	112

The quantity of insertion sites identified are shown in in vitro iPSC and HLC samples infected with SIN’LTR or pHV LV. Insertion site quantity identified in vivo patients infected with HIV (native LTR) or mice (SIN’LTR) are also shown. These insertion sites were mapped to pTFBS using oPossum software and quantity of sites identified are shown.

Examination of the top 10 pTFBS (sequence hits) in infected cells by each vector showed pTFBS mostly in common for iPSC at the two time points of infection (both at 82%) and in transduced HLCs (82%) for each LV. In addition, for each vector, infected iPSC and HLC shared pTFBS at 82% and 100%, respectively. Aligning sites identified between the different cell types at day 3 also showed pTFBS in common for each LV (82% for the SIN’LTR LV and 100% for pHV LV) (Fig. 2). These data confirmed IS choice alignment to be highly similar regardless of cell type, stage of development and LV LTR configuration.

### pTFBS identified in LV LTRs align with vector IS

We next identified the pTFBS in the 5′ LTR sequences of SIN’LTR and pHV via oPOSSUM v3.0 Single Site Analysis (http://opossum.cisreg.ca/). While pTFBS are also found in the promoter region of these viruses, 69% of these sites were not identified in the in vitro data set. A random association of pTFBS in the human genome identified in the Opossum V3.0 database of 170 TFBS families [46] indicated that pTFBS, within a 20 bp window close to the IS chosen by pHV and SIN’LTR in iPSC and HLC, accounted for 25% and 10% of these families, respectively, in the JASPAR database.

**Fig. 3:**
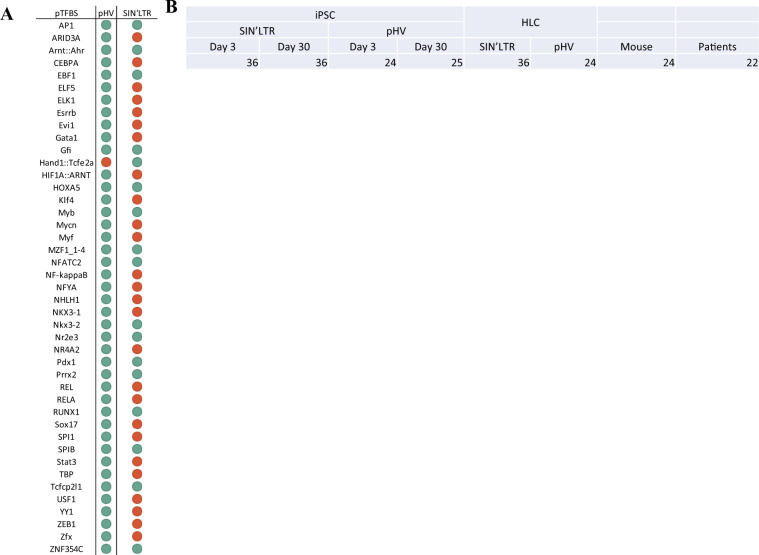
LTR associated pTFBS. **A** Human pTFBS present in 5′ pHV (native) and SIN’LTR vector sequences. Green indicates presence and red indicates absence of pTFBS in the sequence. This indicates 42 sites are identified in pHV, with 17 sites found in SIN’LTR. All sites in SIN’LTR are also seen in the native configuration, except Hand::Tcfe2a. The pTFBS are listed in alphabetical order. **B** Percentage of pTFBS identified with pTFBS found in the LTR. Similar percentages were identified across samples infected with the same LTR configuration. In iPSC day 3 and 30 harvests, was 227,297 out of 633,929 (36%) and 116,539 out of 322,340 (36%), respectively. For pHV, 213,474 out of 329,674 (65%) and 194,721 out of 297,681 (65%) aligned for these cells and time points. In transduced HLC, 44,600 out of 122,760 (36%) and 119,700 out of 185,552 (65%) pTFBS aligned between LTRs and IS, for SIN’LTR and pHV, respectively. The difference in iPSC and HLC alignments was most likely due to differences in the number of integrations between the two cell types.

The frequency of pTFBS common to the SIN’LTR and host IS was 36%. 65% of IS identified were common to SIN’LTR and pHV infected samples Fig. 3. pTFBS were common between each LV regardless of harvest timepoint, cell type and vector copy number.

**Fig. 4:**
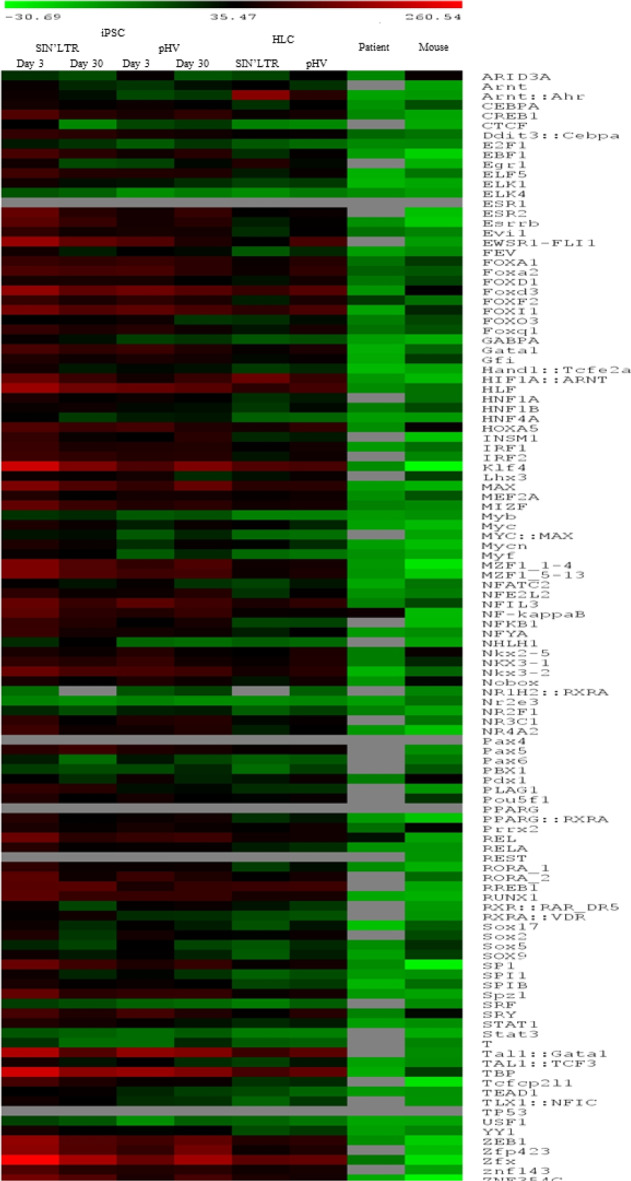
Heat map of Z score enrichment, when compared to background, of species specific pTFBS identified near IS in in vitro iPSC and HLC infected with SIN’LTR or pHV (native LTR) LV, in vivo mouse (SIN’LTR) and HIV patients (native LTR) samples. Enrichment is quantified based on increased presence within 20 bp of the IS compared to presence in a background data set of 100,000 randomly generated 20 bp species specific DNA sequences. A positive *Z* score is taken as enrichment. The pTFBS are listed in alphabetical order. Trends are observed across data sets. Significant enrichment is observed with pTFBS known to be involved in HIV lifecycle. Of 110 pTFBS present in the JASPAR data set, all sites that were close to the IS in SIN’LTR infected iPSC day 3 samples were found enriched and at day 30, 108 of 109 sites were also enriched above background. For pHV transduced iPSC, 108 out of 110 and 109 out of 110 sites were found enriched over background at day 3 and 30 time points, respectively. In HLC transduced by SIN’LTR and pHV LV, 107 of 109 and all 110 sites were enriched above background levels. 17 of 17 and 16 of 17 pTFBS were enriched in SIN’LTR transduced iPSC, at day 3 and day 30 time points, respectively. In pHV transduced iPSC, 41 out of 42 pTFBS in the LTR were enriched for both harvested time points. In HLC, enrichment was identified with 16 of 17 and all 42 pTFBS for cells transduced by SIN’LTR and pHV, respectively 50 out of 112 (45%) of sites were enriched with 9 out of 17 (53%) of pTFBS found in SIN LTR significantly enriched in the mouse.

To determine enrichment of these pTFBS above random expectation, we compared alignments with a randomly generated data set. Calculation of Z scores (enrichment) derived from this analysis showed nearly all pTFBS were significantly enriched above the random data set (Fig. 4).

Compared to the random data set, enrichment of LTR/IS associated pTFBS was proven with positive *Z* scores for most of the pTFBS present in the SIN’LTR and pHV LTRs.

### pTFBS near IS associate with HIV-1 cycle and proliferation

We then examined the TF assigned to each pTFBS and identified HIV-1 based associations. NFATC2, which is known to interact with U3 and U5 to activate HIV-1 genome transcription [47], PBX1 and ZEB1, that have been identified responsible for regulating viral transcription [24] and AP1, which is known to contribute to HIV-1 latency [48]. Also, present was pTFBS known to bind NFκβ and SP1, that are involved in viral gene expression [49, 50]. Interestingly, ZEB1, NFATc2, PBX1, AP-1, NF-kB and SP1 are all associated with cellular proliferation [25–30].

*Z* scores for the binding sites of these TF showed them to be highly enriched above background in iPSC and HLC datasets, suggesting specific targeting of these pTFBS by each LV (Fig. 4). A summary of the enrichments for each pTFBS is shown (Table 2).

**Table 2:** Enrichment data within 20p of IS, as denoted by *Z* scores of species specific pTFBS, of TF known to be involved in HIV lifecycle.

pTFBS	iPSC				HLC		Patient	Mouse
	pHR		pHV					
	Day 3	Day 30	Day 3	Day 30	pHR	pHV		
AP1	60.78	42.3	40.49	48.09	26.54	25.4	−6.63	5.12
NFATC2	40.42	30.47	42.05	22.5	18.65	29.91	−7.73	5.56
NF-κB	118.4	74.52	65.77	84.65	43.64	43.13	50.41	−12.37
PBX1	19.95	16.08	16.7	25.69	7.61	19.87	NI	13.87
SP1	127.3	90.61	61.87	83	42.76	53.05	−0.5	−27.08
ZEB1	163.1	106.5	94.4	119.4	66.92	76.48	−1.21	−17.37

Enrichment is quantified based on increased presence within 20 bp of the IS compared to presence in a data set of 100,000 randomly generated 20 bp species specific DNA sequences. A positive *Z* score is taken as enrichment in comparison to background and are shaded. pTFBS are shown in alphabetical order to observe trends across all data sets. Species specific pTFBSs associated with the HIV lifecycle are enriched across most data sets.

*NI* Not identified.

### Changes in LV associated pTFBS suggests clonal drift

The frequency of pTFBS found in iPSC transduced by SIN’LTR decreased by 49% between day 3 and 30 (from 633,929 to 322,340 sites), in contrast to pTFBS in pHV iPSC, which decreased only 10 % (from 329,674 to 297,681 sites) over the same period. The frequency of pTFBS for TF associated with HIV lifecycle also decreased, however, this was significantly different for each LV (Fig. 2). These differences in pTFBS suggests that, for the pHV vector, retention of these IS could be supporting virus survival. It is possible that this IS preference may also influence outgrowth of cells within pHV bulk populations caused by the native virus LTR promoter and/or enhancer.

### Identification of pTFBS in HIV-1 infected patients IS

To further study the hypothesis that HIV-1 infected cells may exhibit preferential survival compared to their uninfected counterparts as a result of their chosen IS, we investigated pTFBS prevalence near to the IS chosen by HIV-1 in infected patients that had undergone anti-retroviral therapy (ART), which supports the survival of infected cells. We used the Retrovirus Integration Database (https://rid.ncifcrf.gov/intro.php) (RID) to obtain patient IS loci and identified pTFBS (Table 1). These sites were also aligned to those identified in native 5′ LTR. Once again, IS were inputted into BEDTools (https://bedtools.readthedocs.io/en/latest/) to generate 20 bp sequences 5′ and 3′ from each site and map to pTFBS (Table 1) using oPOSSUM v3.0 Single Site Analysis (http://opossum.cisreg.ca/) of these sequences (Fig. 2). Alignment of pTFBS found most sites predicted in the native LTR aligned to patient IS data (39/42). Of the pTFBS alignments for all pTFBS families, most sites (812/1248) were found in common with pTFBS in the HIV LTR and fewer sites (8/42) identified in the LTR were enriched with a positive *Z* score compared to the random data set. pTFBS alignments patient data revealed 28% of pTFBS are enriched, with only PBX1 enriched out of HIV-1 specific TF (Fig. 4).

### Alignment of pTFBS identified in SIN LTR LV and IS in infected mice

We next characterised pTFBS near IS selected by SIN’LTR LV in CD-1 immunocompetent mice (*n* = 31) after gene transfer via yolk sac vessel injection before birth (G16), which efficiently reaches the liver [8]. Following injection, none of the treated mice showed observable adverse effects or tumour development and each displayed normal liver morphology after sacrifice. Four weeks post injection, three mouse livers were harvested for immunohistochemistry of GFP expression and sample DNAs were subjected to LAM PCR [51] followed by BLAST (http://www.ncbi.nlm.nih.gov/genome/seq/MmBlast.html) and BLAT (http://genome.ucsc.edu) searches of the murine genome to determine LV IS (Table 1). From this analysis, out of the IS loci retrieved and pTFBS mapped, all pTFBS present in the SIN LTR aligned (Fig. 2). A similar percentage of the LTR pTFBS was identified in the in vitro iPSC and HLC data sets described in this study.

Using *Z* score significance, 45% of sites were enriched with >50% of of pTFBS found in SIN LTR significantly enriched in the mouse compared to a random control dataset generated using murine background sequences (Fig. 4). This further supports the hypothesis that the LV LTR influences IS choice.

### Alignment of pTFBS found by in vitro and in vivo analyses

By comparison of pTFBS identified in vitro and in vivo, all pTFBS identified in HIV-1 patient IS aligned with those associated with pHV infected iPSC and HLC. In addition, in SIN’LTR infected mice, virtually all pTFBS (97–98%) aligned with pTFBS identified from IS in iPSC and HLC infected with this vector at both time points of infection.

When compared to the random control datasets, as indicated by a positive *Z* score, pTFBS were found to be enriched. In the mouse, these sites included PBX1, NFATC2 and AP1 but not ZEB1, NFκβ and SP1 near IS (Fig. 4). Conversely, in HIV patient IS only pTFBS for NFκβ was significantly enriched. By comparison of Z scores, pTFBS in LV LTRs and IS were highly enriched for TF known to be associated with HIV lifecycle,. Although pTFBS in AP1 and NFATC2, also associated with the HIV lifecycle were moderately enriched, half of these sites were enriched across all data sets (Fig. 4). In iPSC and HLC sites and in mice and patient data sets, NK3-1, PDX1 and PRRX2 were also significantly enriched. AP1, GFI, HOXA5, NFATC2, PBX1, SPIB and NFκβ were also enriched in iPSC and HLC datasets and the murine in vivo data set. Overall, the majority of pTFBS identified in the LV LTRs or those associated with HIV lifecycle are significantly enriched in vitro and in vivo suggesting that the pTFBS in the LTR to be highly associated with in vitro and in vivo data sets and implies infection profiling in iPSC/HLC recapitulates our findings in vivo.

### Alignment of pTFBS in LV IS genes associated with clonal outgrowth

**Fig. 5:**
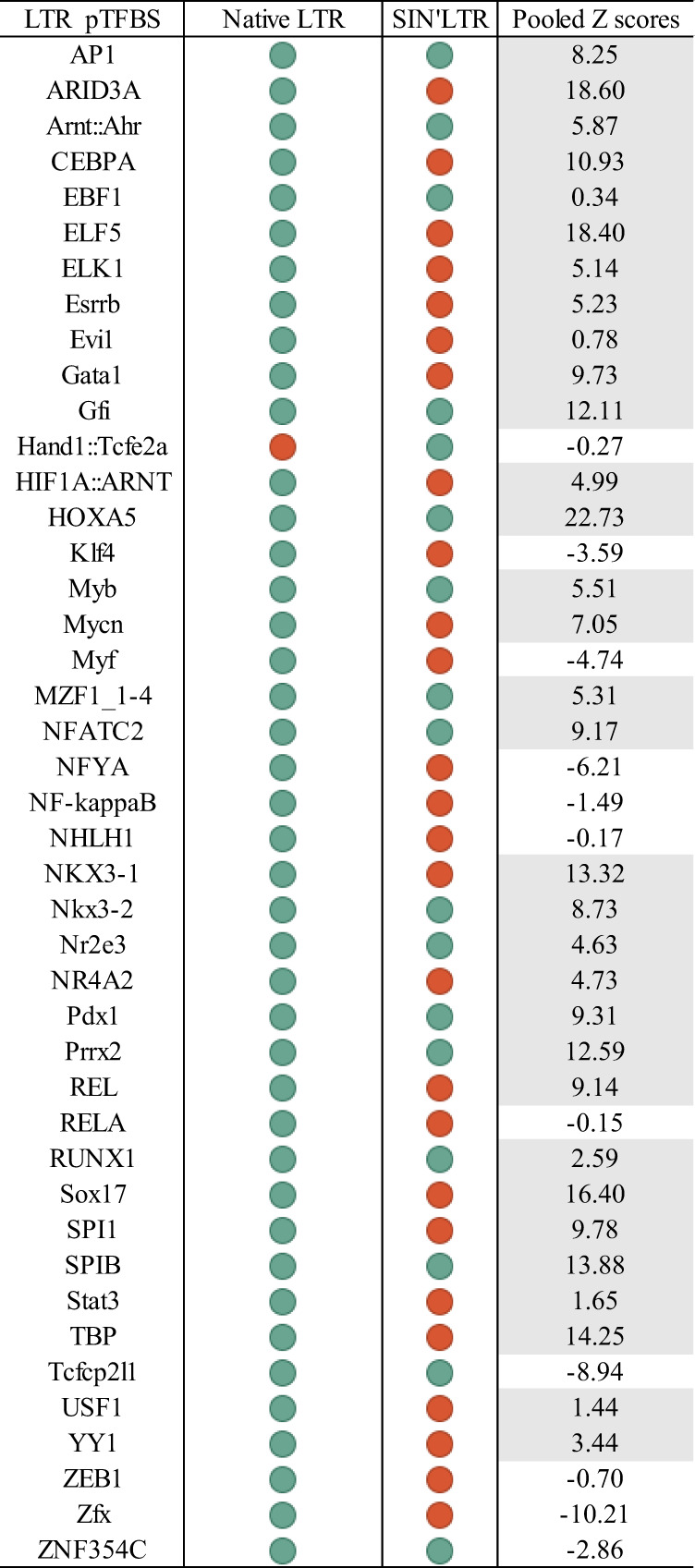
Enrichment of pTFBSs identified in the native or SIN LTR near to *LMO2*, *PRDM16*, *CCND2*, *MECOM*, *HMGA2*, *BMI1*, *BCL2* and *PRDM1* genes. Presence or absence within the LTR configuration are shown as green or red respectively. These eight genes have been previously reported be involved in insertional mutagenesis events. Enrichment is quantified based on increased pTFBS presence in comparison to presence in a data set of 24,752 genes stored in the oPOSSUM database. A positive *Z* score is taken as enrichment in comparison to background and are shaded. The pTFBS are listed in alphabetical order. The enrichment scores have been pooled for all eight genes together. Enrichment is seen for the majority of pTFBSs.

Lastly, we determined whether the pTFBS identified in this study are similar to the IS found near to genes previously reported to be involved in clonal outgrowth following gene therapy [52, 53]. The eight genes we investigated were: *LMO2*, *PRDM16*, *CCND2*, *MECOM*, *HMGA2*, *BMI1*, *BCL2* and *PRDM1* were subject to oPOSSUM v3.0 Single Site Analysis (http://opossum.cisreg.ca/) in which 109 pTFBS were identified. pTFPS present in the native and SIN configuration LTRs were present in all eight genes investigated. Interestingly, the unique pTFBS found in SIN’LTR (Hand::Tcfe2a) was also present in each gene. Enrichment analysis of these pTFBS in these genes against 24,752 genes stored in the oPOSSUM database showed the majority in the native LTR (76%) and the SIN LTR (82%) enriched (Fig. 5) confirming each pTFBS resides in genes known to have been involved in genotoxic events and that the unique pTFBS present in the SIN LTR configuration used in these reported cases, but not the native LTR vector was identified in all eight genes.

## Discussion

HIV IS selection has been studied in several non-clinical models and from patient data from gene therapy clinical trials. LV are known to preferentially integrate into active transcription units in the host genome [4, 5, 7, 54] and various studies have shown insertional mutagenesis after HIV-1 mediated gene therapy [52]. 27.7% of the human genome have been associated with TFBS with 40% of the human genome accessible to TF and, therefore, we sought to investigate the likelihood that TF may influence vector genome tethering using pTFBS common to both the LV IS and the HIV-1 LV native or SIN LTR [55]. TFBS are functionally active and are driven by sequence homology to bind to TF for gene activation [56]. We next identified the importance of the genes chosen for integration and with safety in mind asked whether these genes have been found previously associated with genotoxic events [55].

RV and LV integration requires cleavage of host DNA by the virus integrase, then insertion of the vector and completion of this process by the cellular machinery for successful and permanent residence into the host genome. The tethering model of integration has previously been reported as a mechanism where cell derived proteins chaperone the virus PICs specifically to their chosen target site [54]. DNA repair proteins such as hRad1, are also important to this process by repairing the nicks made in host DNA by the virus integrase [57, 58]. A major protein believed to be involved in tethering is PSIP1/LEDGF/p75, through its interaction with PICs [11, 12]. Interestingly, knockout studies involving PSIP1/LEDGF/p75, show, albeit reduced, preferential integration still into active transcription units, suggesting alternative factors may be supporting genome target site selection [13]. Indeed, whilst interaction between PICs and nuclear import proteins have been shown to be important for efficient entry into the nucleus, these proteins are believed to assist tethering of HIV genomes towards actively transcribing chromatin residing near the nuclear periphery [54].

The virus integrase also shows site specific selection as observed by MLV insertion preference in or close to promoter regions, in contrast to gene transcription units by LV, which is why RV is believed to have a higher risk than LV in causing unwanted insertional mutagenesis through altering gene expression of important genes that regulate normal cellular behaviour [59]. Confirmation of this is evidenced by changing HIV LV site selection to gene promoters though switching of gag/pol sequences between MLV and LV [5].

Studies involving yeast mediated bait selection of proteins have shown that the MLV integrase interact with several proteins that include TF [60]. It has been shown that pTFBS identified in the U3 region of MLV LTR are also present close to IS chosen by these vectors in the host genome, using a search window 1 kb either side of the site of insertion and supports the hypothesis that these sites may also in some way be involved in vector tethering. Interestingly, in that study, pTFBS found in the native HIV-1 LTR were also present near to the IS of infected CD34 + cells, however, in HeLa cells pTFBS IS association could only be identified for MLV and but not LV. However, swapping the U3 region of the LV LTR with that of the U3 of MLV returned this association. This suggests that differences in tethering to pTFBS may be influenced by differential TF gene expression between transformed and untransformed cells [15].

In addition to vector integration site choice, consideration has been made for safer vector design. Both RV and LV carry virus LTRs that reside at the 5′ and 3′ ends of the vector and have promoter and enhancer functions. To circumvent gene activation by these activities, most important to safe LV design has been the development of LTR self-inactivation during reverse transcription. SIN configuration abrogates promoter and enhancer gene activation by U3 deletion. Replication defective vectors with SIN LTR configuration, believed to reduce the potential for insertional mutagenesis [61, 62], are currently promising LV for permanent gene transfer. However, surprisingly these vectors have still been found associated with high frequency oncogenesis in mice and were also implicated in clonal outgrowth in a β-thalassaemia gene therapy trial [63].

To study the tethering model and its possible relationship with IS choice further, we took advantage of the differences in LTR design to determine what effects this had on IS selection by LV in relation to pTFBS. To do this, we used a narrow 20 bp window to investigate for the presence of pTFBS around the IS chosen by native and SIN LTR configurations and aligned these to pTFBS identical sites in each LV LTR. By determination of pTFBS frequency occurring in each LTR with host IS choice we aimed to provide further evidence supporting the hypothesis that TF mediate LV tethering. In addition, we investigated these pTFBS specific sites for their functional properties with regards to HIV-1 and whether these sites are present in genes known to have been targets for insertional mutagenesis.

As we suspected modified cell lines could display gene expression profiles different to untransformed cells and this could influence IS selection, we used human iPSC and their HLC derivatives for our investigation. By using iPSC and their HLC derivatives we expected integration in highly transcribed genes at the early proliferative and the late terminally differentiation stages as would be expected for in vivo or ex-vivo gene transfer in early progenitor and mature cells. These cells have also been used widely for human disease modelling [31–33] and iPSC have also been shown successful in ex-vivogene therapy mediated disease correction [34–36]. The heterogenous nature of cell populations we observed in HLC spheroids may influence LV IS targeting according to differences in gene expression. Interestingly, gene expression in iPSc derived HLC has previously been found to more closely representative of the in vivo microenviroment of the human liver than in liver cell lines [40]. Both iPSc and HLC were characterised as pluripotent stem cells and their differentiated counterparts, respectively, prior to infection and appeared to retain these properties morphologically after infection.

Compared to the randomly generated control datasets, we found pTFBS significantly enriched around LV IS, thereby strengthening the hypothesis that pTFBS present in the LTR either directly or indirectly are involved in LV genome tethering to specific IS. Indeed, this preference appeared to be independent of each cell type used and differences in LTR configurations. Our analysis of pTFBS in these cells was also highly comparable to the pTFBS identified around the IS of HIV-1 in patient genomes carrying native LTR and consistent with our data analysis from in mice injected with the SIN LTR configuration LV.

Interestingly, our pTFBS IS analysis in vitro and in vivo found 50% of LV LTR associated pTFBS around IS involved in HIV-1 lifecycle (NKX3-1, PDX1, PRRX2, AP1, Gfi, HOXA5, NFATC2, SPIB and NFκβ) that appear enriched across most data sets. For example, PBX1 is known to be involved with viral transcription [20–22]. ZEB1 and AP1 have been shown to be involved in HIV latency [24, 48] and NFATC2 is essential for productive infection of non-activated T-cells [23]. Also, NFκβ and SP1 sites in the LTR have both been shown to be involved in transcription of the HIV genome [49, 50]. Interestingly, the majority of these TF have also been previously found associated with cancer [25–30]. We observed that the frequency of pTFBS in these genes decreases over the 3–30 day time period in iPSC infected by SIN’LTR and a significantly smaller decrease of these pTFBS was observed in pHV infected iPSC over the same period (5% compared to 50%). This finding suggests that LV insertion near to these selected pTFBS may be useful to promote virus survival and highlights the importance of the SIN configuration to reduce the potential for insertional mutagenesis of these genes. LEDGF knockout has been shown to significantly decrease HIV-1 integration [11, 12]. As such, mechanistic data using knockout RNAi or TF CHIP-Seq analysis would be interesting to further investigate these findings.

Previous studies have shown that a reduction in IS heterogeneity in infected cells is observed over periods of long-term cell growth. This has been postulated due to reducing polyclonality and clonal outgrowth caused by insertional mutagenesis by vector influence on specific genes involved in cell proliferation [64]. Several vector and host factors are believed to influence vector-associated side effects and the risk of insertional mutagenesis leading to oncogenesis and IS selection and vector configuration are believed highly important to this [57, 65, 66]. This has been demonstrated by the difference shown between RV and LV (tenfold) to cause cellular transformation [67]. With evidence of enrichment of pTFBS that occurred close to IS for each of the LV vectors, in common to these sites in the vector LTR after infections of iPSC and HLC, we investigated whether these pTFBS also occur in genes already previously reported to be associated with clonal dominance in non-clinical genotoxicity models and clinical trials.

We chose eight genes; *LMO2*, *PRDM16*, *CCND2*, *MECOM*, *HMGA2*, *BMI1*, *BCL2*, *PRDM1*, associated with insertional mutagenesis [52] in which 116 pTFBS were assigned and then aligned these with the pTFBS found in each LV LTR. We identified 67% of the pTFBS occurring in the native or SIN LTR configuration within these genes. Interestingly, while the U3 deletion removed multiple pTFBS, it introduces a new pTFBS site not identified in the native LTR (Hand::Tcfe2a).

This work shows that pTFBS present in the LTR are also present in the sites selected by LV for integration suggesting tethering of LV to these sites within the genome. Furthermore, we propose the iPSC/HLC model would be useful to study LV interactions with the host and the outcome of integration into genes important to cell survival and proliferation.

## Methods and materials

### Vector production and titration

The production of HR’SIN-cPPT-SEW-eGFP-W (SIN’LTR) and its native LTR equivalent (pHV) LV was carried out as previously described [68]. These viruses have previously been used in both cell and animal assays [8, 69–71]. The plasmid carrying eGFP flanked by SIN LTRs have also been sequenced to ensure sequence integrity. Both vectors express eGFP under the internal promoter of SFFV (Fig. 1). Infectious LV titre was calculated as previously reported [72]. Briefly, 2 × 10^5^ HEK293T cells were seeded and incubated at 37 °C, 5% CO2 overnight to adhere. Serial dilutions of virus were prepared and incubated in complete cell culture medium with 5 µg/ml polybrene (Sigma Aldrich, Dorset, England), for 20 min at room temperature before addition to cells. 72 h post transfection, cells were harvested for GFP expression analysis via flow cytometry using ACEA Novocyte flow cytometer and NovoExpress software V1.2.5 (Agilent Technologies, Didcot, England). Dilutions expressing 1–30% GFP expression were analysed as accurate representations of viral titre, calculated as shown below:

$${{{{{{{\mathrm{Titre}}}}}}}}\left( {{{{{{{{\mathrm{TU}}}}}}}}/{{{{{{{\mathrm{ml}}}}}}}}} \right) = \left( {\left( {{{{{{{{\mathrm{Cell}}}}}}}}\,{{{{{{{\mathrm{count}}}}}}}} \ast \left( {{{{{{{{\mathrm{Percentage}}}}}}}}\,{{{{{{{\mathrm{GFP}}}}}}}}\,{{{{{{{\mathrm{expression}}}}}}}}/100} \right)} \right)/{{{{{{{\mathrm{Volume}}}}}}}}} \right) \ast {{{{{{{\mathrm{DF}}}}}}}}$$

SIN’LTR LV titre was calculated as 1.18 × 10^9^ TU/ml and pHV was titrated as 3.8 × 10^9^ TU/ml.

### Injection of immunocompetent mice and immunohistochemistry of mouse livers to determine gene delivery

Neonatal MF-1 mice were injected intravenously via the temporal vein 1 day after birth with 4.8 × 10^7^ vector particles/neonate with the SIN’LTR vector to reach their circulation, as previously described. After 72 h, mouse liver samples were harvested via liver biopsy, as previously described [68]. Briefly, each liver biopsy was fixed in 25% formalin overnight, transferred to 70% ethanol, and processed into paraffin. EGFP was detected by incubation in citrate buffer with rabbit anti-eGFP antibody A-6455 (Molecular Probes, Eugene, Oregon, USA). Standard avidin-biotin peroxidase and diaminobenzidine treatment followed and sections were counterstained with haematoxylin. DNA from non-fixed infected mouse tissues and uninfected controls was harvested for analysis of vector integration sites.

### iPSC culture, differentiation, characterisation, and transduction

mTeSR^™^1 medium (Stemcell Technologies, Cambridge, England) was prepared for iPSC growth according to manufacturer’s instructions and stored at 4 °C for further use. 6 and 12 well tissue culture treated plates were coated in 5 µg/well laminin-521 (Stemcell Technologies) as a matrix for stem cell attachment, according to manufacturer’s instructions. Laminin- 521 coated plates were sealed and stored at 4 °C for further use. In preparation for stem cell plating, laminin-521 coated plates were warmed at 37 °C for 20 min.

JHU106i (P106) cells are a hiPS cell line derived from the blood of a 28-year-old Caucasian male, reprogrammed using episomal vectors [73]. These cells were purchased from WiCell, DB41285 (Madison, Wisconsin, USA). These iPSC were grown in mTeSR^™^ 1 -medium and passaged regularly when 70–80% confluent. Morphologically differentiated cells were manually cleared through aspiration. Cells were passaged for at least 1 month prior to initiating differentiation to HLCs to ensure pure cultures of pluripotent stem cells. These cells have been fully characterised against pluripotency and differentiation markers by immunocytochemistry and qPCR analysis [42, 43]. iPSCs were also stained against pluripotent markers and analysed by flow cytometry, namely SSEA4 (96.25 ± 3.75%), TRA-1-60 (95.15 ± 2.35%), TRA-1-81 (88.95 ± 3.75) and against a differentiation marker, CD15 (13.08 ± 3.23%) to verify pluripotency. Cells were washed in DPBS and incubated in gentle cell dissociation reagent (Stemcell Technologies) for 6 min at 37 °C before aspiration of medium. Cells were resuspended in mTeSR^™^ 1 medium and serially diluted between laminin-521 coated wells.

iPSCs were differentiated to HLC in three dimensional spheroid culture as previously reported [42]. The number of cells seeded per microplate were kept consistent at 3.84 × 10^5^ to ensure formation of 256 spheroids of 100–150 µm in diameter form. Endoderm differentiation medium was initiated when the cells were ~30–40% confluent. The culture media was replaced with endoderm differentiation medium RPMI 1640 containing 1 × B27 (Life Technologies, Hemel Hempstead, England) supplemented with essential growth factors, 10 ng/ml Activin A (PeproTech, Hammersmith, England) and 50 ng/ml Wnt3a (R&D Systems, Abington, England). The medium was changed every 24 h for 72 h and then continued with 10 ng/ml Activin A without Wnt3a for 2 days. On day 5, endoderm differentiation medium was replaced with hepatoblast differentiation medium, and this was replaced every 2 days for a further 5 days. The medium consisted of Knockout-DMEM, knockout serum replacement, 0.5% Glutamax, 1% non-essential amino acids, 0.2% b-mercaptoethanol and 1% DMSO (Life Technologies).

Hepatocyte maturation of the iPSCs-derived hepatoblasts was induced at day 10 of differentiation. Cells were cultured using serum-free HepatoZYME^™^ medium (Life Technologies) containing 1% Glutamax (Life Technologies), supplemented with 10 ng/ml hepatocyte growth factor (PeproTech) and 20 ng/ml Oncostatin M (PeproTech), for 13 days. The medium was replaced every 48 h and cells were characterised, as previously described [42, 74]. Cells were analysed at specific time points throughout differentiation using qRT-PCR detection of Oct4, NANOG, FOXA2, HNF4A, SOX17, AFP and albumin protein quantification [42]. Immunohistochemistry against NANOG, OCT4, SOX17 AND FOXA2 (day 0–10), AFP and HNF4A (day 20–30) and E-cadherin (day 30) characterise these cells as PSCs and HLC respectively [42].

Bulk cultures of iPSC and HLC were transduced at MOI 20 using SIN’LTR or pHV LV, using 5 µg/ml polybrene (Sigma Aldrich, Dorset, UK). Bulk cultures were chosen for this analysis as they represent a large population of cells rather than independent clones which may vary in their data outreads. Infected cells remained >85% viable after infection and no morphological changes were observed. Cells were grown for a total of three or serially passaged for 30 days before harvesting DNA and RNA using DNEasy and RNEasy mini kits (Qiagen, Manchester, England) according to manufacturer’s instructions. Cells were analysed via flow cytometry for GFP fluorescence expression and live images of cells were taken under green fluorescence were taken using the Floid^®^ Cell Imaging System (Thermo Fisher Scientific, Hemel Hempstead, England). Infected iPSCs were quantified by flow cytometry to express 90% GFP and HLC spheroids were viewed under fluorescent microscopy to show 85% GFP expression in comparison to brightfield images (Fig. 1). Percentage infection was estimated through observation of all 256 spheroids per microplate after live cells fluorescent microscopy imaging in comparison to brightfield images, as determined by cells expressing green fluorescence.

For analysis of pTFBS, total insertion sites (ISs) from iPSC and HLC samples (three individual bulk samples were grown and differentiated from a single batch of JHU106i cells, in parallel) transduced using LV carrying native (pHV) or SIN configuration (SIN’LTR) LTRs (Table 1) were used for BLAT (http://genome.ucsc.edu) alignment to the human genome build 38 (http://genome.ucsc.edu).

### Vector insertion site analysis

Amplification of vector-genomic DNA junctions: Mouse genomic DNA was extracted as previously described [15, 51]. LAM-PCR: linear amplification for LV vectors was also performed as previously described [44, 45]. Briefly, LAM-PCR of genomic DNA was performed using 100 ng of genomic DNA. PCR products were isolated and cloned into a TOPO TA plasmid cloning kit (Invitrogen) as per manufacturer’s instructions. HIV-ISs were sequenced by deep parallel pyrosequencing (GS FLX/454: Roche, Mannheim, Germany) then subjected to Blas2Seq and the Smith-Waterman algorithm as previously described [75]. Sequences were aligned with the mouse genome (*Mus musculus* genome) assembly (NCBI37/mm9, UCSC *M. musculus* genome) using UCSC BLAT genome browser (http://genome.ucsc.edu) or BLAST (http://www.ncbi.nlm.nih.gov/genome/seq/MmBlast.html).

Insertion sites (ISs) in iPSC and HLC were sequenced through EPTS/LM-PCR adapted from previously described [76].

### Retrieval of patient HIV insertion site data

We used the RID (https://rid.ncifcrf.gov/intro.php) search query to download HIV-1 IS loci in Homo sapiens genome build hg19 (Table 1). We then used BEDTools to retrieve the 20 bp sequences upstream and downstream from each IS from genome build hg19.

### Generation of 100,000 random 20 bp sequences from the hg19 human and mm9 mouse reference genomes

To generate statistically significant background sequences for OPOSSUM analysis we used a Perl script and BEDTools to randomly select 100,000 random 20 bp sequences from the hg19 and mm9 reference genome builds [77].

### Detection of pTFBS at integration loci

We used oPOSSUM v3.0 Single Site Analysis (http://opossum.cisreg.ca/) to find species specific pTFBS that where significantly enriched within the genes targeted by HIV-1. pTFBS were searched for using oPOSSUM3 Single Site Analysis v3.0 (http://opossum.cisreg.ca/) for human targets, using default parameters with all vertebrate profiles with a specificity of 8 bits (minimum), 0.40 conversion cutoff and 85% matrix score threshold. The TF targets identified in oPossum have been shown separately to be validated using CHIP-Seq and therefore are used for reliable identification of pTFBS [78]. 20 bp upstream and downstream of IS were chosen for analysis as this fall within the range of TF binding [79]. Target sequence hits were quantified through filtering for ≥1 hit per transcription factor name identified (Table 1). pTFBS present in 5′ SIN and native LTR configurations were identified through inputting 5′ sequences in a similar fashion [78, 80, 81].

Statistical analysis using *Z* scores were determined using Opossum v3.0 software using species specific background data sets. The *Z* score is determined through comparison of predicted binding sites in the target input set to background set provided. This determines rate of pTFBS found in comparison to expected rate determined from background set to indicate significance of sites found. Positive *Z* scores were used to indicate enrichment of sites through predisposition to sites after LV transduction.

## Data Availability

The databases generated during an/or analysed during the current study are available from the corresponding author on reasonable request.
